# Combined effect of cover crops and bio-fertilizer on sustainable popcorn maize production

**DOI:** 10.3389/fpls.2023.1250903

**Published:** 2024-01-16

**Authors:** Vesna Dragičević, Milena Simić, Željko Dolijanović, Snežana Đorđević, Milovan Stoiljković, Ivica Dimkić, Milan Brankov

**Affiliations:** ^1^ Group for Agro-ecology and Cropping Practices, R&D Department, Maize Research Institute “Zemun Polje”, Belgrade, Serbia; ^2^ Department of Cropping Technology and Agroecology, Faculty of Agriculture, University of Belgrade, Belgrade, Serbia; ^3^ Agrounik d.o.o, Šimanovci, Serbia; ^4^ Laboratory of Physical Chemistry, Vinča Institute of Nuclear Sciences, Belgrade, Serbia; ^5^ Cahir of Biochemistry and Molecular Biology, Faculty of Biology, University of Belgrade, Belgrade, Serbia

**Keywords:** popcorn productivity, popcorn biomass, chlorophyll, grain quality, mineral elements, soil microorganisms

## Abstract

Cover crops play an important role in low-input cropping systems, increasing the use of agro-ecosystem services. Due to the lack of information about the impact of cover crops and bio-fertilizers on popcorn maize (*Zea mays everta* Sturt.) growth and yield quality, especially the popping volume and nutritive quality, such as concentrations of protein and mineral elements, this research aimed to provide essential information. The interrelation between popcorn maize productivity and quality with important groups of soil microorganisms presents additional novelty. The results demonstrated that field pea is a beneficial cover crop, especially when combined with a bio-fertilizer, supporting the accumulation of maize biomass, chlorophyll, yield potential, and the concentrations of protein, Ca, Mg, Fe, and Zn. In addition, field pea residues promoted N-fixing bacteria, and the number of total microorganisms, especially actinomycetes and decomposing bacteria, which could promote nutrient uptake and grain quality. Residues of cover crop mixtures, common vetch + winter oats and field pea + winter oats, promoted the total number of microorganisms in the soil, and up to the end of vegetation, a greater number of decomposition and ammonification microorganisms were found, especially when the bio-fertilizer was applied, which consequently could support greater maize biomass. Popping volume, as a main trait of popcorn maize, had the highest value in the common vetch + winter oats variant, supporting again the statement that quality traits could be enhanced in sustainable production. Unlike living cover crops, mulch mainly affected soil microbial communities and promoted the development of actinomycetes and cellulolytic microorganisms during the growing season. The results of this research could contribute to the development of sustainable popcorn maize production for improved grain quality. They could also serve as a basis for isolating beneficial soil microorganisms to develop new bio-fertilizers that could improve maize production in synergy with cover crops.

## 1 Introduction

Sustainability and regeneration are becoming the main driving forces in today’s agriculture. Practices that support the reduction in agro-chemicals inputs, with greater utilization of the agro-ecosystem services, are welcomed and required to be promptly incorporated in various cropping systems. From this standpoint, cover crops (CCs) could play an important role in agro-ecosystem preservation and regeneration. In addition to the prevention of soil erosion, weed, and pest control (Simić et al., 2020), their role in soil improvements is well recognized through an increase of organic matter content and N retention, thereby decreasing N leaching and greenhouse gas (GHG) emission (White et al., 2017; Abdalla et al., 2019). Irrespective of overall benefits, Abdalla et al. (2019) indicated some disadvantages of CC through the decrease of the yield of the main crop, requiring management that adapts to the specific agro-climatic conditions. Thus, CC from various genera could be successfully used, exhibiting the full effect on soil fertility and main crop yield and fitness. Marcillo and Miguez (2017) indicated that legumes, as well as a mixture of legumes and grasses CC, resulted in greater grain yield when maize was grown on low N or in zero tillage, and what is important, CC tended to stabilize maize yields over time. Some authors (Wortman et al., 2012; Kramberger et al., 2014) underlined better results for main crop productivity and N absorption when CC mixtures were used. In regard to a mixing strategy, Finney et al. (2016) suggested that, in addition to the increasing biomass, the C/N ratio is an important factor in ecosystem services. In general, the use of CC could provide direct and indirect economic benefits through reduction of production costs (fertilizers, pesticides, etc.) and improvements of agro-ecosystem, particularly soil (Daryanto et al., 2018; Jacobs et al., 2022).

Crop productivity and agro-ecosystem improvements could be also supported by bio-fertilizer (BF) use. According to Malusà et al. (2016); Roychowdhury et al. (2017), and Mącik et al. (2020), BFs are recognized among the best tools in modern agriculture that have low environmental impact. They are an unavoidable part of integrated nutrient management to decrease mineral fertilizer inputs, providing an optimal supply of mineral nutrients (P, Ca, Cu, Zn, etc.) to crops. They are also able to fix atmospheric N and deliver it to crops. However, BFs consist of living organisms that come into contact with plant roots, soil particles, and innate communities of soil microorganisms, making it hard to evaluate their efficiency and consistency on crops. Lesueur et al. (2016) urged that high-quality BF must be prioritized when food security and economic advancements are considered. This statement could be supported by the fact that rhizosphere microbiota are variable, dependent on soil characteristics, crop species, phenophase, and a fertilization system (Vollú et al., 2018). From this perspective, it is important to say that CC roots, as well as CC residues, after incorporation into the soil might interact with soil microbiota. Thus, CC could promote mycorrhizal colonization (Njeru et al., 2014), changing the composition and amount of soil microbiota, for cellulolytic activity (incorporation of aerial CC parts) or nitrification activity (roots input) (Gregorutti and Caviglia, 2019).

Popcorn (*Zea mays everta* Sturt.) differs from the common maize types by its popping ability. In some regions, it is an important part of the diet, while in others, it is considered a healthy snack. Popcorn grains are considered a great source of essential amino acids and mineral elements, such as K (up to 2,456 mg/kg), Na (up to 148 mg/kg), Mg (up to 387 mg/kg), Ca (up to 306 mg/kg), and P (up to 2486 mg/kg), with lesser concentrations of Fe, Mn, Zn, Cu, and Cr (Naji et al., 2013; Abebe et al., 2017). Agro-ecological conditions are the top factor that limits crop productivity and quality, particularly when popcorn maize is considered. Hence, the environment plays a significant role in popcorn maize productivity, weight, and chemical composition of popcorn kernel, as well as an expansion volume of flakes (Sweley et al., 2012). Thus, the greater nutritional value of popcorn maize could present a good background for its growing in a more sustainable way, with systems that incorporate CC and BF (Dolijanović et al., 2016; Ahmed and Hassan, 2021).

There is a lack of research about CC, BF, and particularly their interaction on popcorn maize growth and yield quality, especially when nutritive traits were taken into account, such as concentrations of protein and mineral elements, including the most important trait—popping volume. In this research, as a novelty, we connected growing, yield, and quality traits with important groups of soil microorganisms for soil quality and how they are interrelated and affect popcorn maize productivity and quality. The aim of the research was to identify groups of microorganisms that could be used in combination with proper CC important for popcorn maize growth for improving yield potential and its quality.

## 2 Materials and methods

### 2.1 Trial settings and soil properties

The experiment was established during 2018/2019, 2019/2020, and 2020/2021 at the experimental field of the Maize Research Institute “Zemun Polje” (44°52′N; 20°20′E), according to a split-plot design in four replications. The climate was semi-arid, and soil was slightly calcareous chernozem with 30% silt, 17% clay, and 53% sand. The soil properties, prior to cover crop sowing, are given in **Table 1**. In all the three years, after the winter wheat harvest, soil preparation (plowing and seedbed preparation) was performed, succeeded by the cover crop sowing.

**Table 1:** The soil composition, including the soil organic matter (SOM) and available forms of the mineral elements, prior to cover crop sowing.

	N	pH	SOM	P	K	Mg	Ca	Fe	Zn
	kg/ha		%	kg/ha		mg/kg			
Depth (cm)	0–90	0–30							
2019	69.2	7.2	3.32	20.1	30.1	1,105	2,117	23.9	5.06
2020	61.9	7.3	3.05	18.9	25.1	1,402	2,249	24.1	4.81
2021	69.5	7.2	3.18	17.1	32.5	1,537	1,791	46.4	5.14

The following cover crop variants were used in the experiments: CC1, common vetch (*Vicia sativa* L.); CC2, field pea (*Pisum sativum* L.); CC3, winter oats (*Avena sativa* L.); CC4, fodder kale (*Brassica oleracea* (L.) *convar. acephala*); CC5, common vetch + winter oats; CC6, field pea + winter oats; M, organic mulch; and F, control (fallow). The original seed of the Institute for Forage Crops—Institute of Field and Vegetable Crops from Novi Sad was used for planting in all the three years. The cover crops were sown in the last week of October, in the following quantities: common vetch, 120 kg/ha; field pea, 150 kg/ha; oat, 160 kg/ha; and fodder kale, 15 kg/ha. The mixture ratio between legumes and oats was 70:30. In the experimental variant with organic mulch after-harvest residues of winter wheat, an amount of 10 t/ha was arranged over the soil surface. The size of the elementary plot was 35 m^2^.

The mineral fertilization targeted to provide the main crop (popcorn maize) with 120 kg N/ha, 90 kg P/ha, and 60 kg K/ha. Thus, the total amount of P and K was applied in autumn with monopotassium phosphate fertilizer (0:52:34), while the required N amount was incorporated prior to popcorn maize sowing (urea 46% N), with the following pattern: 120 kg N/ha for non-legume crops and control treatments, 80 kg N/ha for sole legumes, and 90 kg N/ha for mixtures. It was assumed that the remaining 40/30 kg N/ha would be provided by nitrogen fixation during CC cultivation.

CC biomass and winter wheat residues were incorporated in the soil using rotovator TF-145 (FPM Deljanin, Kuršumlija, Serbia) at the end of April each year. After that, half of the elementary plot (17.5 m^2^) was treated with BF–Uniker (containing cellulolytic and proteolytic bacteria strains: *Bacillus megaterium*, *Bacillus licheniformis*, and *Bacillus subtilis*, min 10^6^ cm^3^; producer Agrounik d.o.o, Šimanovci, Serbia) in the amount of 10 L/ha to support the mineralization of crop residues. The main crop, popcorn maize (ZPSC 611*k*; FAO 600), was sown in the first half of May, following an arrangement of 70-cm inter-row space and 22-cm intra-row space (65,000 plants ha-1). Standard cultivation and care measures were performed in accordance with the principles of sustainable agricultural technology, i.e., without pesticide application, while weeds were controlled by hoeing, twice during vegetation, in the last week of June and in the middle of July.

### 2.2 Determination of important groups of soil microorganisms

The variations in the total number of soil microorganisms and the number of physiologically important groups from the maize rhizosphere were monitored using indirect dilution, i.e., sowing of suspension of soil samples on growth media. Soil for microbiological analyses was sampled from bulk rhizosphere, 0–20-cm depth, two times per year: 1) prior to popcorn maize sowing and 2) after maize harvesting, at the end of the vegetation cycle. From each elementary plot, three samples were taken (from the middle and two ends) and mixed, from which the average sample per elementary plot was formed. Soil samples were taken using sterile tools, which were cleaned and disinfected with 70% ethanol after every sampling, and then soil samples were placed into sterile glass bottles and transported to the laboratory, where they were stored in the refrigerator (4°C) within 24 h. After short storing, soil samples were homogenized and cleaned through sieves for plant particles (roots, leaves, stalks, etc.). Samples were added in 95 mL of 0.1% (w/v) sodium pyrophosphate solution and then homogenized for 30 minutes. After dilution from 10^−1^ to 10^−7^, aliquots were placed on appropriate substrates. After incubation at 25°C to 30°C, up to 10 days, units forming colonies were determined (CFU). The number of microorganisms was expressed as CFU/g dry soil.

The following substrates were used for microbial growth: tryptic soy agar (TSA; Oxoid, Basingstoke, Hampshire, UK) for total, gram-positive, and gram-negative bacteria; nutrient agar for the determination of ammonifying bacteria; casein-starch agar (Kuster and Williams, 1964) for actinomycete determination, after adding 50 μg/mL isatin, 50 μg/mL cycloheximide, 5 μg/mL polymyxin B sulfate, and 1 μg/mL penicillin G sodium; starch ammonium–nitrate medium for the determination of amino autotrophic bacteria, which use inorganic N sources (Ardiansyah et al., 2019); nitrogen-free medium for the determination of free N-fixing bacteria (dilution 10^6^) and the method of fertile drops for *Azotobacter* (dilution 10^2^) (Anderson, 1965); cellulolytic microorganisms determined using Waksman–Carey substrate (Waksman et al., 1948); fungus number determined using Czapec Dox medium (CZAPEK DOX AGAR, Product Number C 6095; Sigma-Aldrich Corp., St. Louis, MO, USA). After drying at 105°C in a ventilation oven at 60°C (EUinstruments, EUGE425, Novo Mesto, Slovenia) to determine dry matter content, the number of soil microorganisms was calculated per gram of dry soil.

### 2.3 Determination of biomass and yield parameters of popcorn maize

For biomass analysis, the aerial parts of five plants per replication were sampled (cut) in the full anthesis stage and weighted. At the same time, from the same plants, chlorophyll content was measured using the chlorophyll meter SPAD-502 (Konica–Minolta) at three places on the ear leaf blade. Then, plants were dried in a ventilation oven at 60°C (EUinstruments, EUGE425, Novo Mesto, Slovenia) to determine the dry matter (DM) percentage.

Grain yield (GY) was determined at the end of the vegetative cycle (the first half of October) from the two central rows of each elementary plot and calculated at 14% moisture. The popping volume was analyzed according to the standard procedure (Metric Weight Volume Test (MWVT)) using the apparatus Cretors 2300w (Official Metric Wight Volume Tester, Creators), which performs popping with oil. A standard sample of 250 g of popcorn maize grain was popped in four replications. It presented the volume of popped grains (cm^3^) per weight of the grains (g) prior to popping.

### 2.4 Chemical analysis

Each year, prior to the CC sowing, the soil was sampled, and the chemical composition was instantly analyzed, including the pH, soil organic matter content, and contents of the available macro- and micro-elements (N, P, K, Ca, Mg, Fe, and Zn), which were determined (**Table 1**). Soil pH was determined after extraction (0.5 h) with double-distilled water using a pH meter (pH/ION 735, InoLAB Series, WTW; Xylem Analytics, Weilheim, Germany). Soil organic matter (SOM) was determined using the Walkley and Black (1934) method. Available N was determined using the method of Scharpf and Wehrmann (1975), P was determined using the method of Watanabe and Olsen (1965), and available K, Mg, Ca, Fe, and Zn were analyzed on inductively coupled plasma–optical emission spectroscopy (ICP-OES) (iCAP 7000 Series (dual view); Thermo Scientific, Waltham, MA, USA) using Mehlich 3 solution for extraction (Mehlich, 1984).

The average grain sample (approximately 0.5 kg) was dried in a ventilation oven at 60°C (EUinstruments, EUGE425, Novo Mesto, Slovenia) and then milled on Perten 120 (Perten, Hägersten, Sweden; particle size <500 μm). Protein content was determined using an infrared analyzer (Infraneo, Chopin Technologies, Villeneuve-la-Garenne, France), while the concentrations of Ca, Mg Fe, and Zn were determined after wet digestion with HClO_4_ + HNO_3_ using ICP-OES (Thermo Scientific; iCAP 7000 Series (dual view)).

### 2.5 Statistical analysis

The obtained data were processed by three-way factorial analysis of variance (ANOVA, F test), tailored into a split-plot design with four replicates; *p* < 0.05 and *p* < 0.01 were set as a significance level. Biomass (fresh biomass, dry matter percentage, and chlorophyll content) and yield parameters (grain yield and popping volume), as well as grain composition (concentrations of protein, Ca, Mg, Fe, and Zn), are presented as a mean ± standard deviation (SD). Principal component analysis (PCA) as a dimensionality-reduction method was used for the evaluation of interdependence between cover crops and bio-fertilizer regarding important groups of soil microorganisms (prior to maize sowing and after maize harvest), as well as biomass and yield parameters, and grain composition (figures encompassed the first and second axes). Statistical analysis was performed using SPSS for Windows Version 15.0 (SPSS, 2006).

### 2.6 Meteorological conditions

Each experimental season in the 2019–2021 period was characterized by a total precipitation amount that was close to the 2008–2018 average (**Table 2**), ranging from 298.4 mm (2021) to 366.0 mm (2019). Nevertheless, unequal distribution was present. Irrespective that 2019 was the season with the greatest precipitation amount, the lower values were present in July–August, which was the grain-filling period of maize. A similar situation was also present in April and September 2020, as well as September 2021, with the minimum value achieved in April 2020 (germination and sprouting period), with only 4.7-mm precipitation.

**Table 2:** The mean temperature (°C) and precipitation sum (mm) at Zemun Polje during the maize growing period, 2019–2021.

	Average temperature (°C)				Precipitation sum (mm)			
Months	2008–2018	2019	2020	2021	2008–2018	2019	2020	2021
April	14.5	14.6	14.4	10.7	36.2	51.3	4.7	45.9
May	18.8	15.7	16.9	17.9	73.3	129.6	79.9	73.0
June	22.5	24.2	21.3	23.8	75.8	113.7	125.9	19.5
July	24.5	24.1	23.3	26.7	52.7	31.0	34.8	105.5
August	24.5	25.9	25.2	24.3	38.7	19.8	66.3	38.0
September	19.3	18.6	21.9	21.9	44.9	20.6	16.1	16.5
Average/sum	20.7	20.5	20.5	20.9	321.7	366.0	327.7	298.4

Regarding temperature fluctuations, there were minor differences in average values between experimental years and multi-year averages, where 2021 had the highest temperature on average (20.9°C). When the growing season of popcorn maize was considered, the highest values mainly occurred in July–August (grain filling period) in all the three years, according to the multi-year average. Nevertheless, the highest value at 26.7°C was detected in July 2021, including greater average values of approximately 1.4°C and 0.7°C for August 2019 and 2020, respectively, compared to the multi-year average. Cold period in April 2021 during the germination and sprouting period of maize was present. Thus, meteorological fluctuations during the 2019–2021 experimental period could affect maize productivity since the experiment was realized in dry-farming conditions.

## 3 Results

### 3.1 Maize biomass and grain yield

Maize biomass and grain yield were significantly affected by the influence of year, CC, BF, and their interaction (**Table 3**). Year was also a significant source of variation for DM and chlorophyll content. Nevertheless, BF was significant only for DM variations, while CC and Y × CC were significant for chlorophyll variability.

**Table 3:** ANOVA for analyzed sources of variation (F values): year, cover crop, and bio-fertilizer, and their significance for yield parameters and grain quality parameters of popcorn maize.

Sources of variation	Biomass and yield parameters				Popping volume	Grain quality (nutrient concentration)				
	Biomass	Dry matter content	Chlorophyll content	Grain yield		Protein concentration	Ca	Mg	Fe	Zn
Year	1,074.456^**^	54.32^**^	299.89^**^	38.81^**^	3.33	2,052.24^**^	65,819.08^**^	153,731.06^**^	70,118.32^**^	28,961.96^**^
Cover crop	9.164^**^	2.06	3.41^*^	3.28^**^	31.45^**^	21.40^**^	1,572.83^**^	128.21^**^	603.95^**^	93.63^**^
Bio-fertilizer	16.085^**^	9.75^*^	3.66	31.39^**^	0.19	23.60^**^	263.23^**^	1,659.65^**^	1,435.87^**^	67.34^**^
Y × CC	4.381^**^	1.28	3.04^*^	4.02^**^	3.33	11.19^**^	1,207.67^**^	210.59^**^	522.62^**^	94.04^**^
Y × BF	9.301^**^	1.08	0.29	7.13^**^	3.33	18.68^**^	1,578.80^**^	2,246.11^**^	654.58^**^	60.59^**^
CC × BF	0.858^*^	1.35	0.58	2.48^*^	15.17^**^	13.65^**^	3,884.73^**^	393.95^**^	1,219.75^**^	108.32^**^
Y × CC × BF	1.69^*^	1.02	0.57	2.45^**^	3.33	5.27^**^	2,434.17^**^	276.06^**^	809.55^**^	77.41^**^

Y, year; CC, cover crop treatment; BF, bio-fertilizer.

^*^Significant at the 5% probability level.

^**^Significant at the 1% probability level.

**Figure 1:**
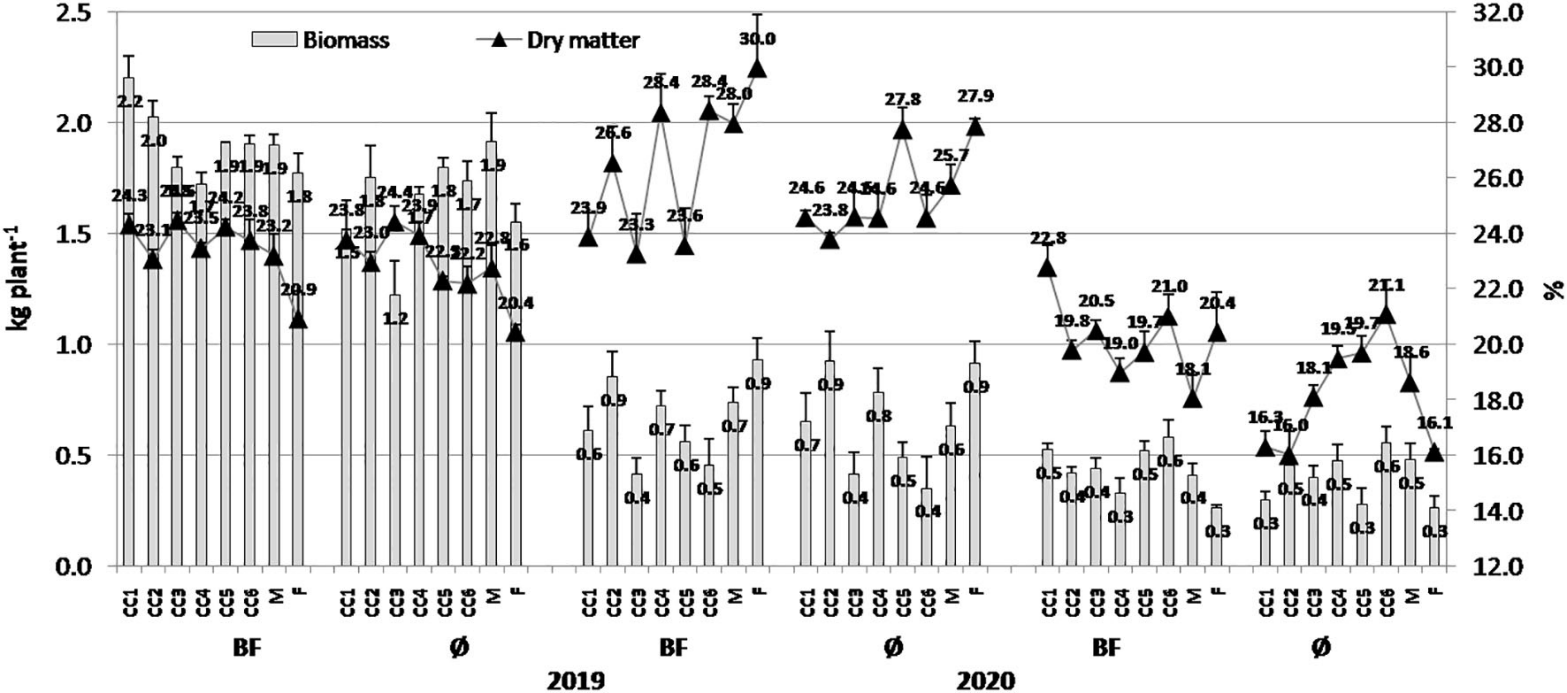
The effect of cover crops (CC1, common vetch; CC2, field pea; CC3, winter oats; CC4, fodder kale; CC5, common vetch + winter oats; CC6, field pea + winter oats; M, organic mulch; F, fallow) and bio-fertilizer (BF) and variant without bio-fertilizer (Ø) on popcorn maize biomass and dry matter content in 2019, 2020, and 2021 (bars represent SD values).

When biomass variations were considered (**Figure 1**), it was obvious that higher values were obtained in 2019, particularly in CC1 (common vetch) + BF combination (2.20 kg/plant), while in variants without BF, the highest value was in CC2 (field pea) treatment (1.75 kg/plant). Lower values were present in general, in 2021, and among variants, greater values were achieved in CC6 (field pea + winter oats) treatments in both combinations, with and without BF (0.58 and 0.56 kg/plant). In contrast to the biomass, DM was greater in general in 2020, with the greatest values of 29.97% (F (fallow)+BF) and 27.88% (F). Again, lower DM values were present in 2021, and among them, greater values were obtained by CC1+BF (22.79%) and CC6 (21.08%). It is noticeable that BF expressed a greater impact on biomass and DM increase in all three experimental years.

**Figure 2:**
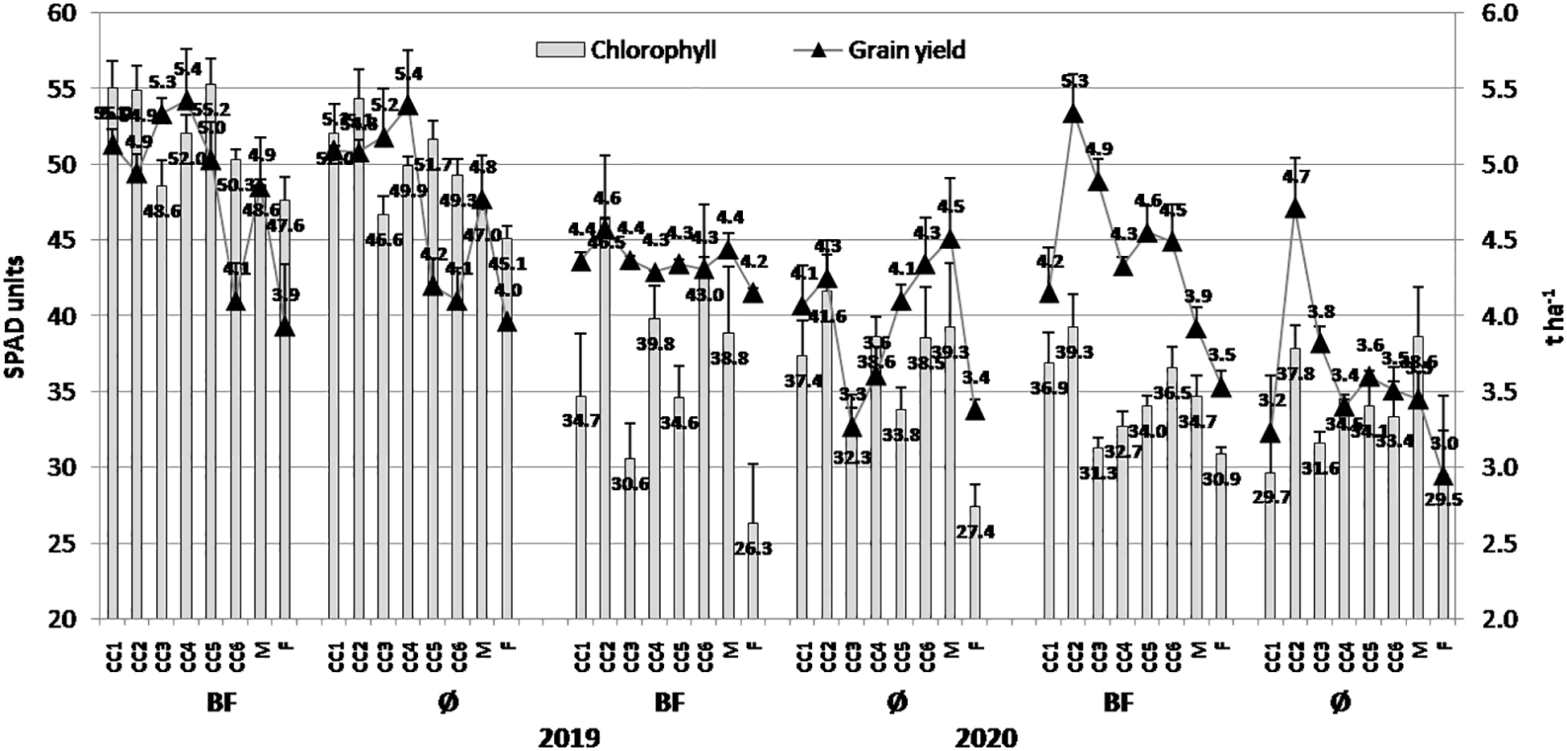
The effect of cover crops (CC1, common vetch; CC2, field pea; CC3, winter oats; CC4, fodder kale; CC5, common vetch + winter oats; CC6, field pea + winter oats; M, organic mulch; F, fallow) and bio-fertilizer (BF) and variant without bio-fertilizer (Ø) on chlorophyll content in maize leaves and grain yield in 2019, 2020, and 2021 (bars represent SD values).

Similarly to the biomass, greater chlorophyll values were present in 2019 in general (55.25 SPAD units and 54.32 SPAD units in CC5 (common vetch + winter oats)+BF and CC2, respectively), while in 2021, lower values were achieved (**Figure 2**). In that year, greater values were in the CC2+BF combination (39.27 SPAD units) and M (mulch) variants (38.63 SPAD units). It is also important to underline that the greater grain yield was mostly present in 2019 (5.42 t/ha in CC4 (fodder kale)+BF and 5.39 t/ha in CC4). The lower values were realized in 2020 and especially 2021, and among them, greater values were in 2020 in the CC2+BF combination (4.57 t/ha) and M (4.51 t/ha), as well as in 2021 in the CC2+BF combination (5.34 t/ha) and CC2 (4.71 t/ha). BF contributed to the realization of greater values of chlorophyll and grain yield in all the three years.

### 3.2 Grain yield quality

Popping volume is an important trait of popcorn maize quality, and it varied significantly under the influence of CC and CC+BF (**Table 3**). The greater impact of all examined factors, year, CC, BF, and their interaction, was present for the concentrations of protein, Ca, Mg, Fe, and Zn in popcorn kernels.

**Figure 3:**
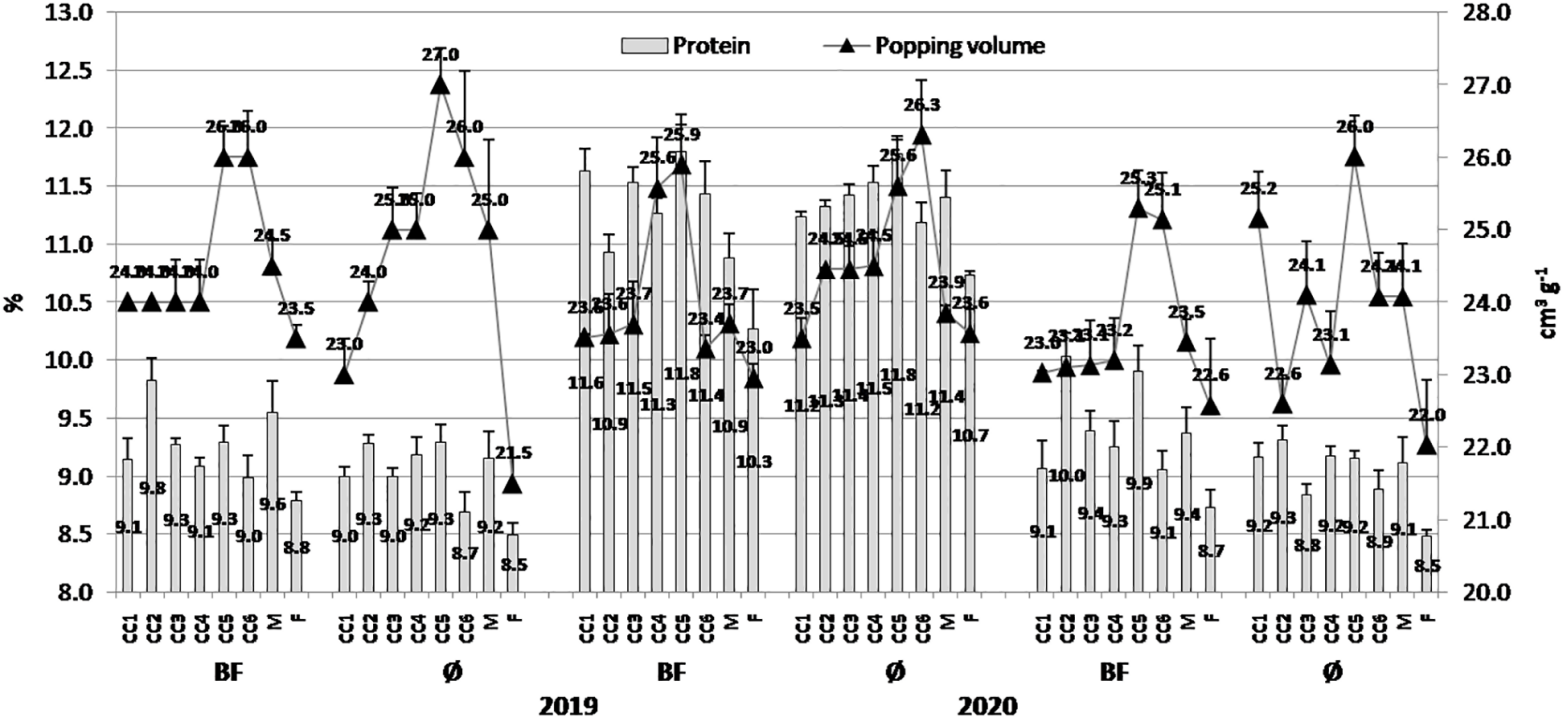
The effect of cover crops (CC1, common vetch; CC2, field pea; CC3, winter oats; CC4, fodder kale; CC5, common vetch + winter oats; CC6, field pea + winter oats; M, organic mulch; F, fallow) and bio-fertilizer (BF) and variant without bio-fertilizer (Ø) on protein content in maize grains and popping volume in 2019, 2020, and 2021 (bars represent SD values).

The general trend was that popping volume was greater in variants without BF application in all the three years (2019, 2020, and 2021) with greater values of 27.0 cm^3^/g (CC5), 26.3 cm^3^/g (CC6), and 26.0 cm^3^/g (CC5), respectively; treatments with BF, greater values were in CC5: 26.0 cm^3^/g, 25.9 cm^3^/g, and 25.3 cm^3^/g, respectively (**Figure 3**). In contrast to the previous statement, greater protein concentration was in treatments with BF application in 2019, 2020, and 2021, with 9.83% (CC2), 11.80% (CC5), and 10.03% (CC2), respectively, while in treatments without BF application, they were 9.28% (CC2), 11.78% (CC5), and 9.32% (CC2), respectively.

**Figure 4:**
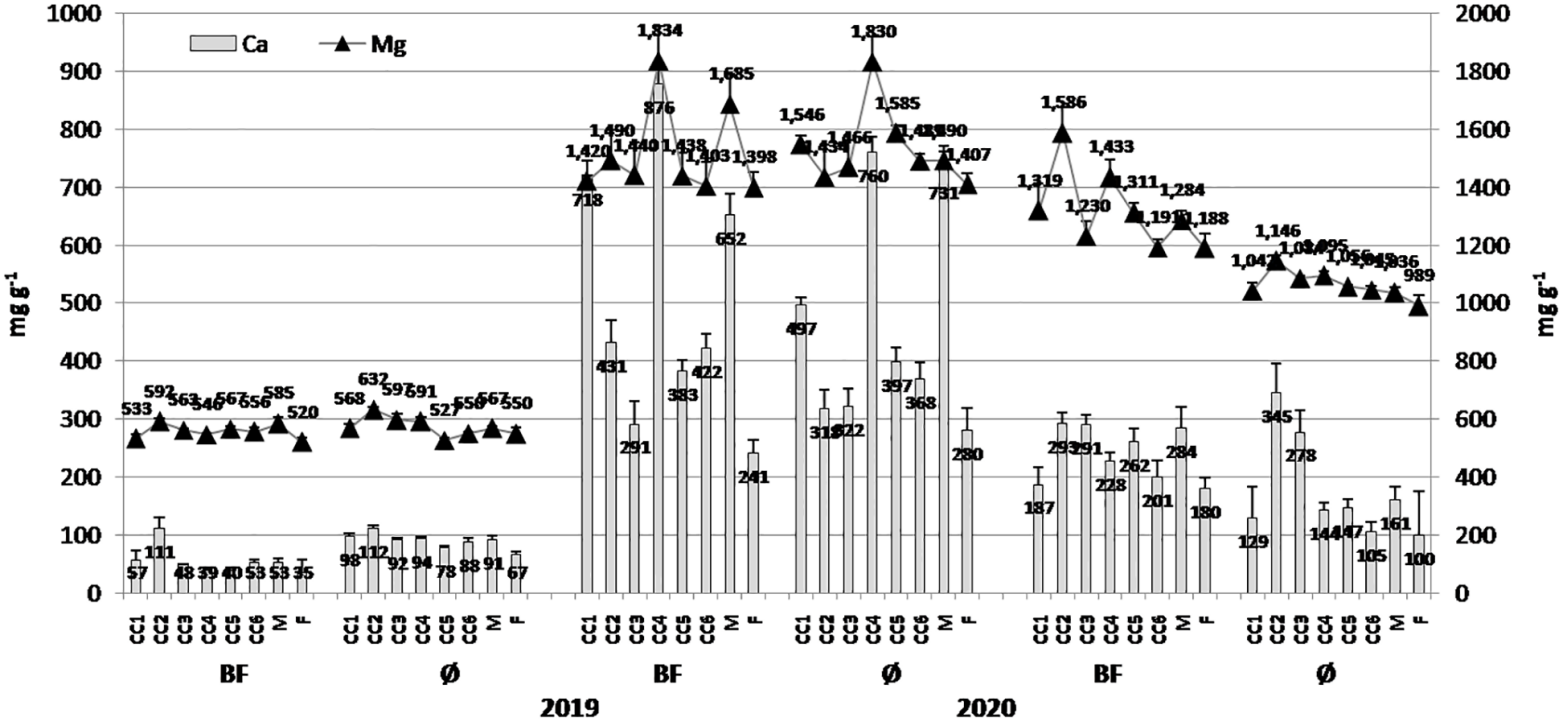
The effect of cover crops (CC1, common vetch; CC2, field pea; CC3, winter oats; CC4, fodder kale; CC5, common vetch + winter oats; CC6, field pea + winter oats; M, organic mulch; F, fallow) and bio-fertilizer (BF) and variant without bio-fertilizer (Ø) on Ca and Mg concentrations in maize grains in 2019, 2020, and 2021 (bars represent SD values).

**Figure 5:**
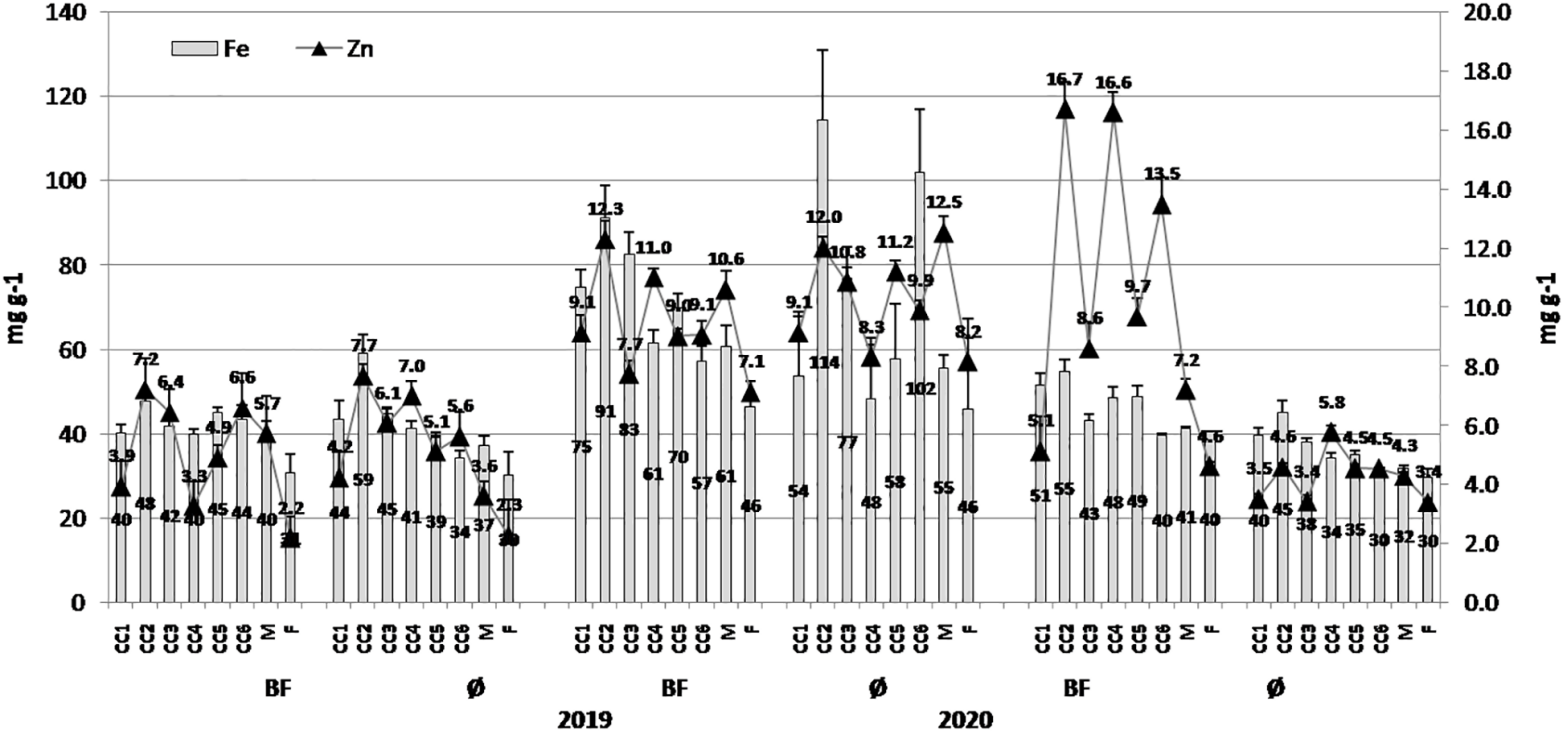
The effect of cover crops (CC1, common vetch; CC2, field pea; CC3, winter oats; CC4, fodder kale; CC5, common vetch + winter oats; CC6, field pea + winter oats; M, organic mulch; F, fallow) and bio-fertilizer (BF) and variant without bio-fertilizer (Ø) on Fe and Zn concentrations in maize grains in 2019, 2020, and 2021 (bars represent SD values).

Essential elements in popcorn kernels varied in greater range over years and applied treatments. In general, the greater values of all examined elements were in 2020: 876.34 µg/g (CC4+BF) and 759.61 µg/g (CC4) for Ca, 1,834.1 µg/g (CC4+BF) and 1,830.2 µg/g (CC4) for Mg (**Figure 4**), and 91.26 µg/g (CC2+BF) and 114.37 µg/g (CC2) for Fe (**Figure 5**). For Zn, the greatest value at 16.71 µg/g was achieved in CC2+BF in 2021 and 12.02 µg/g in CC2 in 2020. In addition to the fluctuations induced by year, BF was an important source of variability, particularly when Ca, Mg, and Zn were considered.

### 3.3 Principal component analysis for CC, BF treatments, and popcorn maize traits

**Figure 6:**
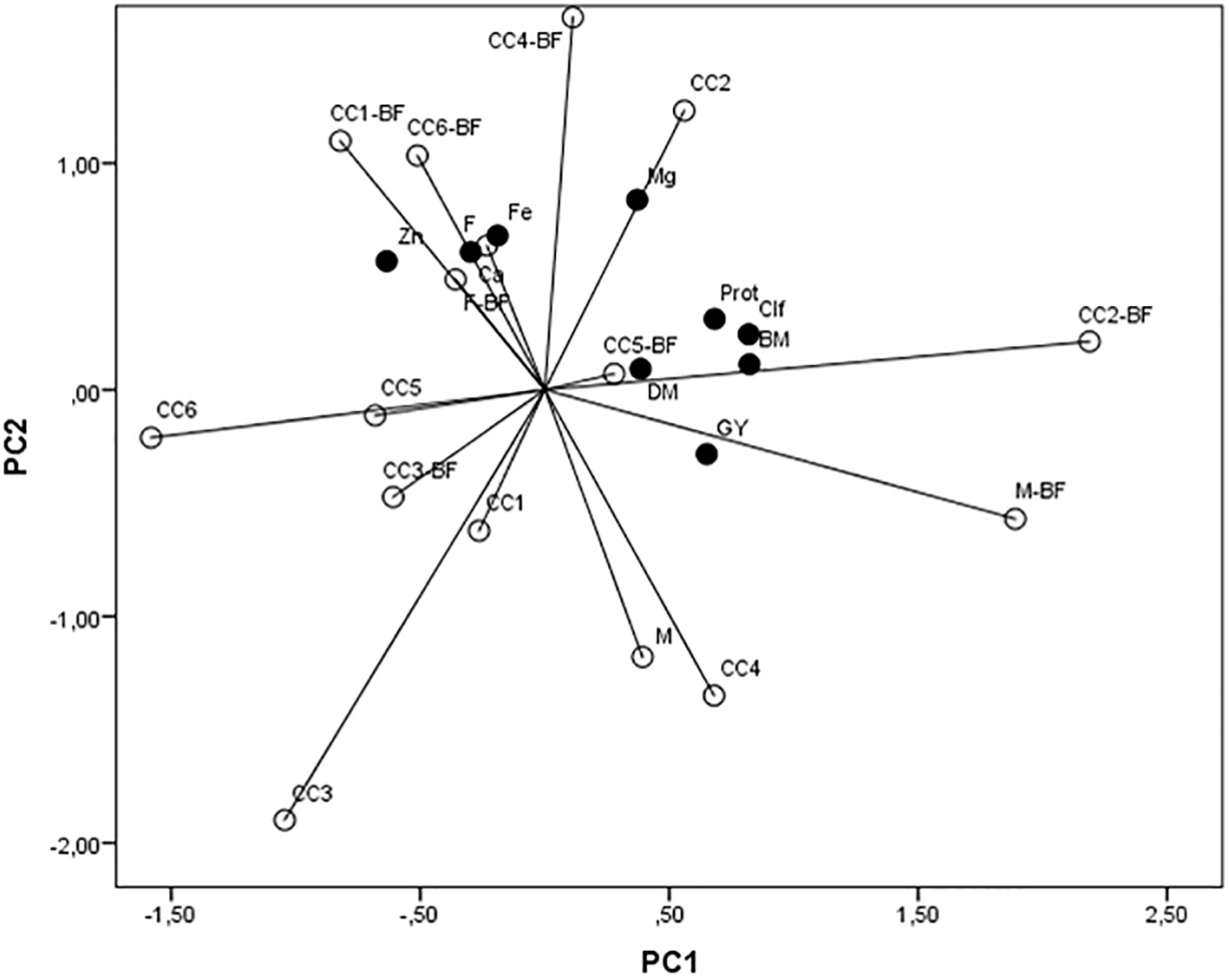
A principal component analysis of the popcorn maize biomass (BM), dry matter content (DM), chlorophyll content (Clf), grain yield (GY), protein content in grain (Prot), and concentrations of Ca, Mg, Fe, and Zn in grain influenced by cover crops (CC1, common vetch; CC2, field pea; CC3, winter oats; CC4, fodder kale; CC5, common vetch + winter oats; CC6, field pea + winter oats; M, organic mulch; F, fallow) and bio-fertilizer (BF).

Interdependence within applied treatments (CC and BF) and maize biomass parameters and grain quality was processed by PCA. The first axis contributed with 33.9% in total variability, the second contributed with 23.6% (**Figure 6**), and the third one contributed with 13.69%. Among examined traits, biomass, chlorophyll, grain yield, and protein concentration correlated significantly and positively with the first axis, while Zn correlated negatively. Ca, Mg, and Fe correlated significantly and positively with the second axis, and DM correlated positively with the third axis. The variations of Ca, Fe, and Zn were mainly present in CC1+BF, CC6+BF, F+BF, and F, while Mg varied mostly in the CC2 treatment. Other traits, such as biomass, DM, grain yield, chlorophyll, and protein concentration, varied mostly under the influence of CC2+BF, CC5+BF, and M+BF.

### 3.4 Variations of the important groups of soil microorganisms

The results in **Table 4** indicate the significant impact of all factors on the variation of important groups of soil microorganisms: year, CC, and their interaction in the spring, before popcorn maize sowing, as well as year, CC, BF, and their interaction in the autumn, after maize harvest.

**Table 4:** ANOVA for analyzed sources of variability (F values): year, cover crop, and bio-fertilizer and their significance for number of important microorganisms (MO) in soil.

Sources of variability	Total MO	Ammonifying MO	Free-living diazotrophs	*Azotobacter* sp.	Casein-starch agar		Fungi	Cellulolytic MO		
					Total bacteria	Total actinomycetes		Bacteria	Fungi	Actinomycetes
Prior to popcorn maize sowing (spring)										
Year	246.71^**^	179.42^**^	745.67^**^	7.79^**^	2,828.54^**^	18.15^**^	18.15^**^	1,572.52^**^	63.08^**^	123.15^**^
Cover crop	1,529.45^**^	2,345.27^**^	128.08^**^	27.85^**^	1,146.51^**^	5.11^*^	5.11^*^	99.97^**^	5.63^**^	5.60^**^
Y × CC	1,042.15^**^	2,926.69^**^	221.25^**^	16.60^**^	1,202.60^**^	14.35^**^	14.35^**^	113.57^**^	5.80^**^	15.44^**^
After popcorn maize harvest (autumn)										
Year	5,830.67^**^	1,743.52^**^	3,376.73^**^	10.67^**^	18,778.86^**^	64,056.84^**^	64,052.75^**^	1,527.36^**^	137.41^**^	19.15^**^
Cover crop	596.92^**^	46.60^**^	250.58^**^	13.88^**^	605.33^**^	893.67^**^	893.62^**^	100.42^**^	16.68^**^	17.64^**^
Bio-fertilizer	4,373.24^**^	464.08^**^	1,439.16^**^	489.99^**^	1,312.35^**^	37.51^**^	37.50^**^	114.15^**^	6.23^*^	21.80^**^
Y × CC	398.22^**^	159.91^**^	287.54^**^	19.35^**^	1,862.25^***^	802.46^**^	802.40^**^	73.60^**^	7.48^**^	20.11^**^
Y × BF	343.05^**^	133.76^**^	544.26^**^	18.32^**^	393.80^**^	24.43^**^	24.43^**^	84.07^**^	8.41^**^	13.66^**^
CC × BF	278.97^**^	60.92^**^	237.28^**^	8.43^**^	69.26^**^	47.58^**^	47.57^**^	21.16^**^	9.02^**^	8.76^**^
Y × CC × BF	149.83^**^	119.39^**^	144.06^**^	8.88^**^	54.93^**^	99.10^**^	99.09^**^	19.46^**^	9.01^**^	7.51^**^

Y, year; CC, cover crop treatment; BF, bio-fertilizer.

^*^Significant at the 5% probability level.

^**^Significant at the 1% probability level.

Based on the average values from all three seasons, in spring, the greater total amount of soil microorganisms and free-living diazotrophs was detected in the CC5 variant (79.4% and 32.5% greater than in fallow, respectively; **Supplementary Table 1**). Ammonifying and cellulolytic bacteria had increased numbers in CC6 (39.3% and 21% higher, in comparison to fallow, respectively), while the number of *Azotobacter* spp. and bacteria developed on casein-starch agar was greater in CC2 (7.9% and 24.3% greater, in comparison to fallow, respectively). The CC1 contributed to the increase in fungus content (13.2% higher in regard to fallow), while all other cover crops and mulch contributed to a significant decrease in fungi prior to sowing. M and CC6 contributed to the increase of actinomycete number (49.9% higher in regard to fallow). Cellulolytic fungi had the greatest number in CC3, up to 50%, in comparison to fallow.

A greater variability in the number of important soil microorganisms was developed up to the autumn, i.e., popcorn maize harvest, where the CC1 contributed to a greater extent to the increase in the number of majority of examined microorganisms, such as total microorganisms, ammonifying microorganisms, free-living diazotrophs, and fungi (39.4%, 19.9%, 38.6%, and 38.7%, in comparison to fallow, respectively; **Supplementary Table 1**), while the CC1+BF contributed mostly to the increase in number of *Azotobacter* spp. (11.8%, in comparison to fallow). The M+BF was also a variant where the greater number of total microorganisms and fungi was found, especially a greater number of cellulolytic bacteria and cellulolytic fungi (26.4% and 28.6%, respectively), while in the M, the greatest number of cellulolytic actinomycetes was present (50% in comparison to fallow). The greatest number of actinomycetes developed on casein-starch agar existed in the CC2 and particularly the CC2+BF (18.4% in comparison to fallow), while the most abundant bacteria developed on casein-starch agar was in CC6+BF (44.8% in comparison to fallow).

**Figure 7:**
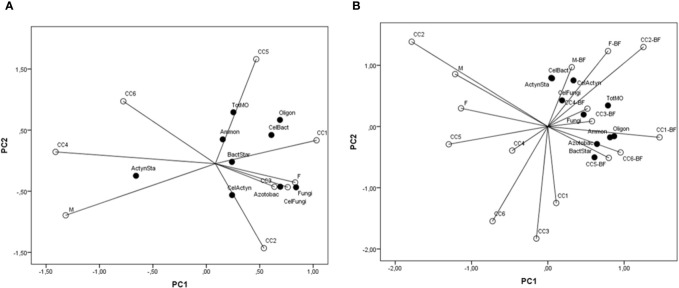
A principal component analysis of the important groups of soil microorganisms (TotMO, total number; Ammon, ammonifying microorganisms; FLD, free-living diazotrophs; Azotobact, *Azotobacter*; CSABact, casein-starch agar total bacteria; CSAActyn, casein-starch agar total actinomycetes; Fungi; CelBact, cellulolytic bacteria; CelFungi, cellulolytic fungi; CelActyn, cellulolytic actinomycetes) influenced by cover crops (CC1, common vetch; CC2, field pea; CC3, winter oats; CC4, fodder kale; CC5, common vetch + winter oats; CC6, field pea + winter oats; M, organic mulch; F, fallow). **(A)** In spring before popcorn maize sowing; **(B)** In autumn after popcorn maize harvest.

When PCA was considered, regarding soil microorganisms in spring, the first axis contributed with 32.5% in total variability, the second with 23.1%, the third with 19.1%, and the fourth with 12.5%. Free-living diazotrophs, *Azotobacter* spp., fungi, cellulolytic bacteria, and fungi positively correlated significantly with the first axis, while actinomycetes developed on casein-starch agar correlated negatively; total microorganisms correlated significantly and positively with the second axis; ammonifying microorganisms and bacteria developed on casein-starch agar correlated positively with the third axis; only cellulolytic actinomycetes correlated positively with the fourth axis. According to the data in **Figure 7A**, actinomycetes developed on casein-starch agar varied mostly in the CC4 and M variants. Ammonifying microorganisms and total microorganisms mainly varied in CC5, bacteria developed on casein-starch agar varied mostly in CC1, and cellulolytic bacteria and free-living diazotrophs varied mainly in both CC5 and CC1 variants. Cellulolytic actinomycetes varied in CC2, as well as *Azotobacter* spp. and fungi, and cellulolytic fungi mainly varied in CC3 and F variants.

Regarding PCA from autumn sampling, the first axis contributed with 32.0% in total variability, the second with 25.5%, the third with 16.0%, and the fourth with 10.1%. Total microorganisms, ammonifying microorganisms, free-living diazotrophs, *Azotobacter* spp., and bacteria developed on casein-starch agar correlated significantly and positive to the first axis; actinomycetes developed on casein-starch agar, cellulolytic bacteria, and actinomycetes correlated positively with the second axis; cellulolytic fungi correlated positively with the third axis, while fungi correlated negatively with the same axis. Data from **Figure 7B** indicate that the greatest variability of cellulolytic bacteria and actinomycetes developed on casein-starch agar was present in M+BF, the greatest variability of cellulolytic fungi was in F+BF variant, and cellulolytic actinomycetes varied greatly in M+BF and F+BF. Total microorganisms and fungi varied mostly in CC3+BF and CC4+BF. The greatest variability of ammonifying microorganisms and diazotrophs was present in CC1+BF, although *Azotobacter* spp. and bacteria developed on casein-starch agar varied greatly in CC5+BF and CC6+BF.

## 4 Discussion

### 4.1 The impact of cover crops on popcorn maize productivity, grain quality, and soil microorganisms

Cover crops are an important part of sustainable agriculture. Their role was recognized in soil recovery and reduction of weed infestation, including broader environmental impacts (Marcillo and Miguez, 2017; Daryanto et al., 2018; Simić et al., 2020), which could lead to an increase in crop productivity and quality over time. In addition, Jacobs et al. (2022) proved that conservation systems, including soil cover over winter diminished soil loss, have also greater a benefit of 43% lesser costs than the conventional system.

In this research, it was proved that cover crops positively affect the growth and yield parameters of popcorn maize. It was shown that field pea (CC2) mainly supported chlorophyll synthesis and biomass accumulation in seasons with relatively optimal meteorological conditions, resulting thus in higher grain yield, while in unfavorable seasons, such as 2021, it contributed to greater grain yield achievement, indicating the importance of leguminous CC, i.e., catch CC, for maize productivity (Santos et al., 2019). Marcillo and Miguez (2017) specified that maize yield could be increased up to 33%, after leguminous CC, when zero tillage was used or N fertilization was low. This is consistent with results obtained in this study; in addition, in drier seasons with higher average temperature, during the grain filling period (2021), grain yield increased after field pea, as CC was 37% greater, in comparison to fallow. The combination of field pea and winter oats (CC6) had a greater impact on maize growing parameters—biomass accumulation and DM percentage during the unfavorable year—supporting the statement of Wortman et al. (2012), who pointed the advantage of mixtures over sole CC due to the greater productivity and possible increased ecological resilience to meteorological extremes. It could be also the reason for the greater grain yield achieved in 2019 after fodder kale as CC, which is well known for forming great biomass and coverage compared with other CCs used in this study.

It is well known that leguminous CCs could improve soil nitrogen balance, decrease N leaching, and increase its continual availability through the vegetation of the main crop (Kramberger et al., 2014; White et al., 2017; Abdalla et al., 2019), thus increasing protein concentration in maize grain. In our research, field pea (CC2) enabled high and stable protein concentration in popcorn maize grain, while in 2020, the season where protein concentration was higher than in 2019 and 2021, common vetch + winter oats (CC5) was superior over other variants, indicating that proper management of CC and soils could exploit all of the CC benefits in N retention in soil and its supply to the main crop (White et al., 2017). Ciampitti and Vyn (2013) emphasized the positive correlation between greater N supply to maize plants and enhanced accumulation of Ca, Mg, and microelements in grain. Thus, the greater protein concentration in popcorn maize grains was followed by a greater accumulation of essential mineral elements, such as Ca, Mg, Fe, and Zn, contributing to the nutritional quality of popcorn grains, which was mainly present in the field pea variant. The increased number of *Azotobacter* spp. and bacteria developed on casein-starch agar and variability in the number of cellulolytic actinomycetes present in the CC2 variant prior to popcorn maize sowing could be connected to the potentially greater availability of N and other essential nutrients, thus supporting maize metabolism, resulting in greater biomass and yield potential, as well concentrations of protein, Ca, Mg, Fe, and Zn, in the grain. In contrast to field pea, common vetch (CC1) was not important for maize biomass, yield parameters, and mineral nutrients absorption, while it contributed to the increase in number of fungi and cellulolytic fungi and also increased variability of cellulolytic bacteria, free-living diazotrophs, and bacteria developed on casein-starch agar in spring. Thus, it could be considered the CC that had a greater impact on soil, but not crop, reducing the availability of nutrients and crop productivity (Kramberger et al., 2014; White et al., 2017). A prolonged effect of leguminous CCs was present up to the harvest of popcorn maize, increasing the number of total microorganisms, ammonifying microorganisms, free-living diazotrophs, fungi, and cellulolytic bacteria. Irrespective that field pea increased Mg variability in popcorn maize grain, in a similar study, Dragicevic et al. (2021) indicated fodder kale as CC that enhanced Mg concentration in sweet maize kernels, while common vetch + winter oats contributed to the greater Zn accumulation and winter oats to the greater Fe accumulation.

When quality was considered, mixtures such as common vetch + winter oats (CC5), followed by field pea + winter oats (CC6), were also important for greater values of popping volume, which is the most important trait of popcorn maize. The same treatments were also essential for increasing the number and variability of total microorganisms, ammonifying microorganisms, free-living diazotrophs, cellulolytic bacteria, and fungi in the spring. While greater maize biomass and DM percentage were achieved particularly in field pea + winter oats (C6) (during unfavorable seasons), the positive impact of CC mixtures on important microbial groups and the increased popcorn productivity and quality was underlined, presenting a novelty of this research.

The importance of mulch (M) was emphasized during unfavorable seasons, supporting chlorophyll synthesis (2021) and yield potential (2020). This variant contributed a greater number and variability of actinomycetes developed on casein-starch agar in spring, and cellulolytic microorganisms, particularly cellulolytic actinomycetes to the autumn, evidencing the importance of cellulolytic microorganisms and actinomycetes to maize growing potential, in general. Rodrigues et al. (2016) indicated that millet, as a gramineous CC, contributed to the increase in actinomycete populations as important nutrient cyclers. A similar trend was observable in the M variant, but not in winter oats (CC3). It must be mentioned that Chen et al. (2023) showed that the incorporation of different straw types—from wheat or maize, irrespective that both are Poaceae crops—has distinct effects on the fungal and bacterial communities in soil.

### 4.2 Contribution of BF to popcorn maize productivity, grain quality, and changes of soil microorganisms

Bio-fertilizers are important in sustainable agriculture, promoting soil quality, crop growth, and yield potential, at the same time (Roychowdhury et al., 2017; Mącik et al., 2020). They are able to promote nutrient absorption, supporting the quality of agricultural crops, with low environmental impact (Lesueur et al., 2016; Malusà et al., 2016; Dragicevic et al., 2021).

In this research, BF alone promoted biomass and DM accumulation only in 2020 (the season with the highest unequal precipitation distribution), while its interaction with CC benefits for popcorn maize growth, yield potential, and quality were recognized. Thus, greater variability in concentrations of Zn, Fe, and Ca was present in neither F+BF and CC1+BF nor the mixture CC (field pea + winter oats)+BF variants, supporting the synergistic effect of BF and CC. Thus, BF could be a tool to improve maize grain quality through enrichment with mineral nutrients, similar to the results previously obtained on sweet maize (Dragicevic et al., 2021). Singh et al. (2013) also emphasized that BF in combination with green manure is able to improve soil and crop quality at the same time. In addition, the CC1+BF and CC6+BF variants also supported the increase and variability of the important soil microorganisms, such as free-living diazotrophs and *Azotobacter* spp. (CC1+BF), as well as ammonifying microorganisms and bacteria developed on casein-starch agar (CC6+BF), which were followed by a greater accumulation of biomass and dry matter (particularly present during favorable season), indicating the importance of these groups of microorganisms for maize biomass development, as well as maize ability to absorb essential nutrients. Jalal et al. (2020) and Aasfar et al. (2021) confirmed that diazotrophic bacteria (in combination with various N sources) are able to promote soil quality and thus contribute to greater maize growth and yield potential. Similarly, Njeru et al. (2014) revealed greater mycorrhizal colonization of maize roots after hairy vetch (*Vicia villosa* Roth) and CC mixture, which resulted in greater biomass and N and P concentrations in aboveground parts of maize.

Moreover, the BF combination with field pea (CC2) contributed mainly to the chlorophyll accumulation and increased grain yield values in relatively unfavorable seasons, such as 2020 and 2021, supporting crop fitness. When stressful conditions were considered, Anli et al. (2020) showed that inoculation with mycorrhizal fungi and selected consortia of plant growth-promoting rhizobacteria, together with organic fertilizers, enhanced the resilience of date palms to drought stress, also increasing the productivity. Nevertheless, the most important effect of CC2+BF was present in a greater accumulation of protein and essential elements (Ca, Mg, Fe, and Zn), even higher than in the CC2 sole variant. Thus, the CC2+BF supported variability of biomass, grain yield, accumulation of DM, and chlorophyll, as well as protein in grains, in general, whereas the lesser variability was present in the combination of BF + common vetch + winter oats (CC5) and BF + mulch (M). Similar research indicated fodder kale and common vetch + winter oats + BF as the top CC combinations for essential elements accumulation in sweet maize kernels (Dragicevic et al., 2021). It is also important to underline that after popcorn maize harvest, a greater number of actinomycetes developed on casein-starch agar present in the soil of the CC2+BF variant, such as in CC2 sole variant, indicating their potential importance for maize growth and yield quality, particularly when availability of mineral elements was considered (Sathya et al., 2016). Due to the fact that actinomycetes are known to boost legume growth and quality (AbdElgawad et al., 2020), it could be assumed that the prolonged effect of field pea residues enhanced actinomycete development and activity, resulting in improved nutrient availability and thus maize growth and yield quality.

Irrespective that M+BF did not express a greater impact on maize growth, yield potential, and quality, its impact on soil microbial community was pronounced, through increased number and variability of fungi and cellulolytic microorganisms (bacteria, fungi, and actinomycetes) up to autumn. Gregorutti and Caviglia (2019) detected that aerial crop residues from wheat (*Triticum aestivum* L.) and white sweet clover (*Melilotus albus* Medik.) when incorporated into soil promoted activity of cellulolytic microorganisms, while root incorporation promoted nitrification activity. In that research, whole residues (stalks + roots) were incorporated in the soil, so it could be assumed that the greater cellulose amount was incorporated in the M variant than in variants with living CCs (C1–C6), giving an advantage to cellulolytic microorganisms over time, i.e., to autumn. Breza-Boruta and Bauza-Kaszewska (2023) underlined the synergistic effect of microorganisms from bio-fertilizers and organic matter in soil on microbial communities, indicating variability in crop response to bio-fertilizer application and soil status. The prolonged effects of CCs and CCs+BF were not covered in this study, which could present the potential for further research, with a retrospective view on crop rotation where some additional synergic effects could be achieved.

Correspondingly, the synergic effect of CC mixtures, CC5+BF and CC6+BF, was reflected in greater chlorophyll and particularly protein accumulation in popcorn maize grains. Similarly, Janosevic et al. (2017) indicated greater chlorophyll content and especially protein concentration in sweet maize kernels when grown after CC mixtures + BF. Furthermore, CC5+BF was the variant with greater popping volume achieved (slightly lower than in CC5 alone), indicating again that for some traits, CC has a greater impact than CC+BF.

## 5 Conclusion

Since there is little information in the literature about the impact of CC and BF on popcorn maize growth and yield, this research provided new insights into sustainable popcorn maize production for yield and grain quality and its possible connection with soil microbial communities.

The results showed that field pea is a beneficial cover crop, especially when combined with bio-fertilizer, which supports growth parameters (biomass and chlorophyll content), yield potential, and nutritional status by increasing the concentrations of protein, Ca, Mg, Fe, and Zn. In addition, field pea residues were good media for N-fixing bacteria. A sustained effect was obtained by the increased number of total microorganisms, especially actinomycetes and decomposing bacteria, which could promote the uptake and accumulation of minerals and proteins in popcorn maize grain. In particular, the combination of field pea and BF could increase the fitness and resistance of plants in stressful conditions. Residues from cover crop mixtures of common vetch + winter oats and field pea + winter oats promoted the total number of microorganisms in the soil. By the end of vegetation, especially when BF was applied, a greater number of decomposition and ammonification microorganisms were found, which consequently could support a greater accumulation of maize biomass, chlorophyll, and protein in the grain. Popping volume, as a main trait of popcorn maize, had the highest value in the common vetch + winter oats variant (in combination with BF and especially without it), supporting the statement that quality traits could be particularly enhanced in sustainable production, presenting a novelty of this study. Unlike live cover crops, mulch was insignificant for popcorn maize traits but mainly affected soil microbial communities and promoted the development of actinomycetes, as well as benefited cellulolytic microorganisms during the growing season.

The results of this research could support the development of sustainable popcorn maize production for improved quality. Furthermore, the obtained results could also serve as a basis for isolating beneficial soil microorganisms to develop new bio-fertilizers, which synergistically with cover crops could enhance maize production.

## Data availability statement

The original contributions presented in the study are included in the article/**Supplementary Material**. Further inquiries can be directed to the corresponding author.

## Author contributions

VD: experiment design; chemical analysis; statistical analysis; manuscript writing; MSi: experiment design; manuscript writing¸ material and funding acquisition; ŽD: experiment organization; SĐ: experiment design; microbiological analysis; MSt: chemical analysis; ID: manuscript editing; MB: experiment organization; statistical analysis; manuscript writing. All authors contributed to the article and approved the submitted version.
