# Supplementary material for: Combined effect of cover crops and bio-fertilizer on sustainable popcorn maize production
Source: Front Plant Sci. 2024 Jan 16;14:1250903. doi: 10.3389/fpls.2023.1250903 (PMC10824896; doi:10.3389/fpls.2023.1250903)
Supplement: Supplementary file 1 [file DataSheet_1.pdf]

Suppl. Table 1. The impact of cover crop and bio-fertilizer on number of important microorganisms (MO) in soil (three-year average).

Sources of variability		Total MO (CFU × 10^5 g^{-1} dry soil)	Ammonyfing MO (CFU × $10^5 g^{-1}$ dry soil)	Free living diazotrophs (CFU × 10^5 g^{-1} dry soil)	*Azotobacter spp.* (CFU × $10^5 g^{-1}$ dry soil)	Casein-starch agar		Fungi (CFU × $10^5 g^{-1}$ dry soil)	Cellulolytic MO (CFU × $10^5 g^{-1}$ dry soil)		
						Total bacteria (CFU × 10^5 g^{-1} dry soil)	Total actynomycetes (CFU × $10^5 g^{-1}$ dry soil)		Bacteria	Fungi	Actynomycetes
Prior to popcorn maize sowing (spring)											
	CC1	74.00	154.00	95.33	421.67	128.00	0.40	25.33	62.00	2.00	5.33
	CC2	38.00	324.67	73.33	445.00	299.33	0.47	14.00	30.67	2.00	4.67
	CC3	96.67	176.00	72.00	418.33	138.00	0.47	18.00	49.33	2.67	6.00
	CC4	150.00	262.00	72.67	380.00	88.67	0.47	6.67	40.67	0.00	6.00
	CC5	197.33	262.67	112.67	398.33	214.67	0.13	16.00	58.00	2.00	4.00
	CC6	148.67	405.33	92.67	423.33	227.33	0.53	11.33	70.00	0.67	4.67
	M	52.00	162.67	72.00	350.00	182.00	0.53	4.00	40.67	0.00	3.33
	F	40.67	246.00	76.00	410.00	226.67	0.27	22.00	55.33	1.33	6.00
After popcorn maize harvest (autumn)											
Ø	CC1	260.67	284.67	230.00	361.67	147.33	0.75	20.67	48.67	2.00	8.67
	CC2	150.67	180.00	136.67	366.67	96.67	1.24	12.00	81.33	0.67	6.67
	CC3	181.33	257.33	169.33	415.00	98.67	0.44	10.67	44.00	3.33	3.33
	CC4	182.67	279.33	166.67	370.00	62.67	0.79	8.67	69.33	4.00	5.33
	CC5	169.33	215.33	161.33	348.33	102.00	0.64	12.67	72.67	2.00	7.33
	CC6	145.33	238.00	186.67	365.00	118.00	0.58	11.33	49.33	2.00	4.00
	M	208.00	200.67	166.67	366.67	98.00	0.97	12.67	71.33	2.00	12.00
	F	158.00	228.00	141.33	376.67	92.00	1.05	12.67	70.67	3.33	6.00
BF	CC1	260.00	280.00	222.00	465.00	183.33	0.85	11.33	54.67	0.67	5.33
	CC2	300.67	307.33	220.00	423.33	128.00	1.27	14.00	85.33	4.00	8.00
	CC3	232.00	262.00	194.67	458.33	102.67	0.75	12.67	64.67	3.33	9.33
	CC4	288.00	257.33	194.00	441.67	92.67	6.47	12.00	76.67	2.00	7.33
	CC5	216.00	273.33	196.00	436.67	138.67	6.13	10.00	64.00	3.33	6.00
	CC6	166.00	308.00	179.33	448.33	186.00	6.00	13.33	68.00	1.33	6.00
	M	342.00	264.00	171.33	411.67	121.33	9.73	14.67	96.00	4.67	12.00
	F	230.00	302.00	213.33	410.00	102.67	10.33	12.67	70.67	3.33	10.00
